# Diagnosis of Infantile Diseases

**Published:** 1886-11

**Authors:** 


### *DIAGNOSIS OF INFANTILE DISEASES.*

1. Congestion of the cheeks in children, excepting in cases ot cachexia and chronic disease, indicates an inflammation or a febrile condition.

2. Congestion of the face, ears and forehead, of short duration, strabismus with febrile reaction, oscillation of the iris, irregularity of the pupil, with falling of the upper lips, indicate a cerebral affection.

3. A marked degree of emaciation which progresses gradually, indicates some sub-acute or chronic affection of a grave character.

4. Bulbar hypertrophy of the fingers, and curving of the nails are signs of cyanosis.

5. Hypertrophy of the spongy portion of the bones indicates rachitis.

6. The presence between the eyelids of a thick and purulent secretion from the Meibomian glands may indicate great prostration of the general powers.

7. Passive congestion of the conjunctival vessels indicates approaching death.

8. Long-continued lividity as well as lividity produced by a motion and excitement, the respiration continuing normal, are indices of a fault in the formation of the heart or the great vessels.

9. A temporary lividity indicates the existence of a grave acute disease, especially of the respiratory organs.

10. The absence of tears in children four months old or more suggests a form of disease which will usually be fatal.

11. Piercing and acute cries indicate a severe cerebo-spinal trouble.

12. Irregular muscular movements, which are partly under the control of the will during the hours when one is awake, indicate the existence of chorea.

13. The contraction of the eyebrows, together with a turning of the head and eyes to avert the light, is a sign of cephalalgia.

14. When the child holds his hand upon his head, or strives to rest the head upon the bosom of his mother or nurse, he may be suffering from ear disease.

15. When the fingers are carried to the mouth, and there is, beside, great agitation apparent, there is probably some abnormal condition of the larynx.

16. The act of scratching or of pinching the nose in children indicates the presence of worms or of some intestinal trouble.

17. When a child turns his head constantly from one side to another, there is a suggestion of some obstruction in the larynx.

18. A hoarse and indistinct voice is suggestive of laryngitis.

19. A feeble and plaintive voice indicates a trouble in the abdominal organs.

20. A slow and intermittent respiration accompanied with sighs, suggests the presence of cerebral disease.

21. If the respiration is intermittent but accelerated, there is capillary bronchitis.

22. If it is superficial and accelerated, there is some inflammatory trouble of the larynx and trachea.

23. A strong and sonorous cough suggests spasmodic croup.

24. A hoarse and rough cough is an indication of true croup.

25. When the cough is clear and distinct, there is bronchitis.

26. When it is suppressed and painful, there is pneumonia and pleurisy.

27. If the cough is convulsive, it indicates whooping cough.

28. Sometimes one sees a dry and painless cough in the course of typhoid and intermittent fever, in the course of difficult dentition, or an attack of worms, under these conditions the cough is often due only to a bronchitis which has been caused by the original disease.—Dr. Bradley, (*L' Union Med. du Canada.*)

